# Optimized Method for *Pseudomonas aeruginosa* Integrative Filamentous Bacteriophage Propagation

**DOI:** 10.3389/fmicb.2021.707815

**Published:** 2022-01-12

**Authors:** Damir Gavric, Petar Knezevic

**Affiliations:** Department of Biology and Ecology, Faculty of Sciences, University of Novi Sad, Novi Sad, Serbia

**Keywords:** filamentous phage, *Pseudomonas* phage Pf4, *Pseudomonas* phage Pf5, *Pseudomonas* phage PfLES, plaque, phage propagation, phage purification, ssDNA

## Abstract

Filamentous bacteriophages frequently infect *Pseudomonas aeruginosa* and alter its phenotypic traits, including virulence factors. The first step in examination of these phages is to obtain suspensions with high virus titer, but as there are no methods for integrative filamentous phage multiplication, the aim was to design, describe, and compare two methods for this purpose. As models, three strains of *Pseudomonas aeruginosa*, containing (pro)phages Pf4, Pf5, and PfLES were used (PAO1, UCBPP-PA14, and LESB58, respectively). Method 1 comprised propagation of phages in 6 L of bacterial culture for 48 h, and method 2 applied 600 mL culture and incubation for 6 days with centrifugation and addition of new medium and inoculum at 2-day intervals. In method 1, phages were propagated by culture agitation, followed by centrifugation and filtration (0.45 and 0.22 μm), and in method 2, cultures were agitated and centrifuged several times to remove bacteria without filtration. Regardless of the propagation method, supernatants were subjected to concentration by PEG8000 and CsCl equilibrium density gradient centrifugation, and phage bands were removed after ultracentrifugation and dialyzed. In the obtained suspensions, phage titer was determined, and concentration of isolated ssDNA from virions was measured. When propagation method 2 was compared with method 1, the phage bands in CsCl were much thicker, phage number was 3.5–7.4 logs greater, and concentration of ssDNA was 7.6–22.4 times higher. When phage count was monitored from days 2 to 6, virion numbers increased for 1.8–5.6 logs, depending on phage. We also observed that filamentous phage plaques faded after 8 h of incubation when the double layer agar spot method was applied, whereas the plaques were visible for 24 h on single-layer agar. Finally, for the first time, we confirmed existence of replicative form and virions of PfLES (pro)phage as well as its ability to produce plaques. Similarly, for the first time, we confirmed plaque production of Pf5 (pro)phage present in *P. aeruginosa* strain UCBPP-PA14. The described method 2 has many advantages and can be further improved and adopted for filamentous phages of other hosts.

## Introduction

Bacteriophages have come into focus as potential antibacterial agents during the last two decades. For more than a century, they have been used in eastern European countries for medical treatment of bacterial infections, whereas in the rest of the world antibiotics were used almost exclusively (Zaczek et al., 2020). Nowadays, as infections with multi- and pan-drug resistant bacteria have become a worldwide threat, interest in phage therapy, and thus phage biology, have significantly increased. Tailed bacteriophages are appropriate for phage therapy, so the majority of known phages are tailed representatives (more than 95%) belonging to order *Caudovirales* (Ackermann and Prangishvili, 2012). As a consequence, methods for tailed phage propagation, concentration, and purification are well established (Oślizło et al., 2011; Bennett et al., 2012; Ali et al., 2019).

An insufficiently examined group of phages, commonly known as filamentous bacteriophages, belongs to the order *Tubulavirales* (Knezevic et al., 2021). These phages possess helical symmetry of coat subunits around circular positive single stranded DNA (+ssDNA). They release from a cell by extrusion without killing the host and usually without plaque production, impairing the phage isolation and propagation. Even if they form plaques, they are usually turbid and considered to appear as a consequence of decreased bacterial growth rather than cell lysis. However, on the lawn of some hosts, regular plaques can be formed. Many of these phages can integrate DNA into the host genome and replicate by rolling circle mechanism with replicative form (RF) as extrachromosomal circular DNA as a template for replication. The most extensively examined phage from this order belongs to species *Escherichia virus M13* (family *Inoviridae*), which are used as cloning vectors in the phage display and nanotechnology (Rakonjac, 2012). These phages are not able to integrate into the bacterial chromosome, persist in a form of a plasmid and produce plaques. Green and Sambrook (2017) recommend propagation of M13 bacteriophage in a liquid culture, and it is possible to obtain a high titer of virions in supernatant (∼10^12^ PFU/mL). However, many phages belonging to family *Inoviridae* are not so productive, i.e., their production rate is significantly lower, particularly if they integrate DNA into bacterial chromosome (Lerner and Model, 1981; Rakonjac, 2012; Knezevic et al., 2021). For new or less examined filamentous phages, the appropriate host for their isolation/propagation is unknown, and in addition, many potential hosts are already infected by other filamentous bacteriophages, interfering with propagation of a target phage strain. The most reliable method for their propagation is cultivation of strains that are naturally infected with a phage of interest in a liquid culture to allow spontaneous phage production. For instance, *P. aeruginosa* strains PAO1, UCBPP-PA14, and LESB58 are infected with (pro)phages designated as Pf4, Pf5, and PfLES, respectively, so during bacterial strain cultivation, Pf phages are constantly produced. It is confirmed that Pf4 produces plaques and a medium virus titer (Webb et al., 2004), whereas Pf5 is produced in a lower titer, and plaque production is not reported (Mooij et al., 2007). There is no data about PfLES virion production and plaque formation.

Pf1 related (pro)phages are very frequent among *P. aeruginosa* strains as their genetic elements are detectable in more than 60% of *P. aeruginosa* strains (Knezevic et al., 2015). They can contribute to the mobile DNA content of their hosts, influence microbial population dynamics in different environments and cause a short-term evolution (García et al., 2019). Filamentous phages are also involved in *P. aeruginosa* phenotypic variability, including influence on adhesion, biofilm formation and dispersion (Webb et al., 2004; Secor et al., 2016a), antibiotic sensitivity (Hagens et al., 2006), formation of small colony variants (SCV; Rice et al., 2009), bacterial motility (Bucki et al., 2015; Secor et al., 2016b), and pyoverdin production (Manos et al., 2008; Yeung et al., 2009). Moreover, these phages enhance *in vivo* virulence (Rice et al., 2009) and contribute to chronicity of infections (Burgener et al., 2019). Taking into account the filamentous phage prevalence and influence on *P. aeruginosa*, further examination of this bacteriophage group is very important. The first step in such studies is usually phage propagation and purification, but there is no optimized protocol for this purpose.

Here, we described two methods for propagation of *P. aeruginosa*–specific filamentous bacteriophages to obtain higher phage yield for their further characterization.

## Materials and Methods

### Bacterial and Phage Strains

In the study, three *P. aeruginosa* strains: PAO1, UCBPP-PA14, and LESB58, naturally infected with filamentous (pro)phages Pf4, Pf5, and PfLES are used, respectively (Table 1). The strain TuD43, which does not possess any filamentous Pf1 prophage genetic elements and shows susceptibility to all three phages, was used for phage titer determination. The strains were maintained as glycerol stocks at −80°C and grown overnight in Luria-Bertani (LB) broth at 37°C.

**TABLE 1:** *Pseudomonas aeruginosa* strains used in the study.

Strain designation	Genome sequence access. no.	Prophage designation	Prophage coordinates	(Pro)phage genome size (bp/bases)	References
PAO1	NC_002516.2	Pf4	785311–797747	12,437	Webb et al. (2004)
UBCPP-PA14	NC_008463.1	Pf5	4345126–4355800	10,675	Mooij et al. (2007)
LESB58	NC_011770.1	PfLES	4545190–4555758	10,569	Knezevic et al. (2015)
TuD43	N.A.	N.A.	N.A.	–	–

*N.A., not available.*

Because there is no proof that PfLES can be produced, we designed a primer pair to confirm existence of its RF based on previously predicted prophage coordinates in the LESB58 genome (Knezevic et al., 2015). The primers 5′-ACGGCTCTGCACTTCTACG-3′ and 5′-CGACAGTTCTTCGACACTTGC-3′ were used for colony PCR of LESB58 with the following parameters: initial cycle of 94°C for 5 min followed by 35 cycles of 94°C for 30 s, annealing at 53°C for 20 s, and extension at 72°C for 60 s with a final 7 min extension at 72°C. For the PCR, DreamTaq Green master mix (2x) was used (Thermo Fisher Scientific, Lithuania). The PCR product was sequenced by the dideoxy method, and the sequence was deposited in NCBI. As a positive control, we used the primer pair for *P. aeruginosa* RpoD (Savli et al., 2003) and universal Pf phage primer pair PfUb (Knezevic et al., 2015). In addition, as a negative control for PfLES phage RF was used PAO1 strain. The agarose gel electrophoresis (0.7%) was applied to detect PCR products and documented (BioDocAnalyze System, Biometra, Germany).

### Methods for Phage Propagation

#### Propagation Method 1

Method 1 comprised inoculation of 6 L (six flasks with 1 L of culture; a total volume of flask 2 L) of LB broth with 1 mL overnight culture and 48 h incubation at 37°C with agitation (200 rpm). After the incubation, bacterial cultures containing phage particles were centrifuged (10,000 × *g*, 10 min) and filtered (0.45 and 0.22 μm). The phages from resulting supernatants were precipitated overnight at 4°C with polyethylene glycol 8000 (PEG8000) and NaCl in final concentrations of 4% and 0.5 M, respectively. The following day, phage suspensions were centrifuged at 12,000 × *g*, for 15 min at 4°C, supernatant was decanted, and the tubes were inverted and allowed to dry. The resulting phage precipitate was dissolved in 2–3 ml of SM buffer (the original volume was concentrated 2000–3000 times).

#### Propagation Method 2

For method 2, each *P. aeruginosa* strain was inoculated with 1 mL of overnight culture in 600 mL of LB broth (in flask of a total volume 1 L) and incubated at 37°C with constant agitation (200 rpm) for 2 days. After incubation, bacteria were removed from medium containing phages by centrifugation at 10,000 × *g* for 10 min. The centrifugation step was repeated until there was no more bacterial residue (approximately three times). The resulting supernatant was harvested in a new vessel and amended with sterile medium to the starting volume (600 mL). The medium containing phages was again inoculated with 1 ml of overnight bacterial culture and further incubated for 48 h. The abovementioned steps were repeated on the fourth day, and on the sixth day, only centrifugation steps were applied to obtain phage suspensions without bacteria.

**FIGURE 1:**
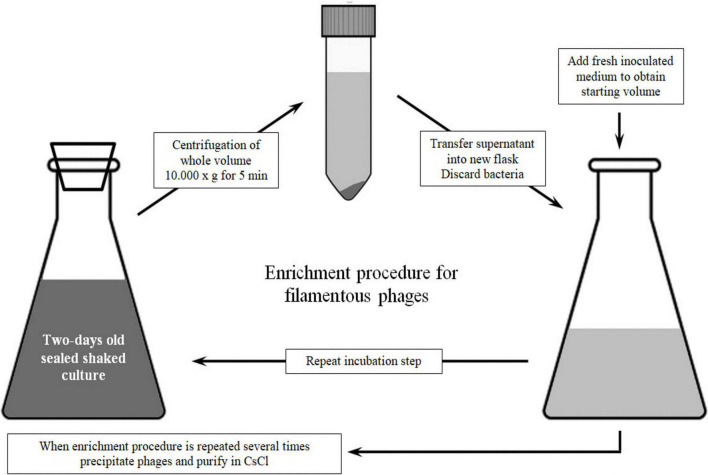
Schematic representation of method 2 for Pf phage propagation.

Subsequently, bacteriophages were precipitated with 4% PEG8000 and 0.5 M NaCl. The tubes were kept on ice at 4°C overnight, and then the phages were pelleted at 12,000 × *g*, 4°C for 15 min, and the resulting supernatant was decanted. As the phage precipitate stayed on sides of tube walls, the centrifugation step was repeated once more for 5 min to bring precipitate at the tube bottom. After drying, the phage pellets were collected in 6 mL of SM buffer by vortexing (the original volume was concentrated 100 times). The schematic presentation of method 2 with key steps is shown in Figure 1.

### Filamentous Phage Titer Increases Over Time

Bacterial cultures were incubated for 6 days as for the propagation method and a small amount of phage suspension was separately precipitated with PEG8000 (40 ml) after 2-day intervals and resuspended in 500 μl of SM buffer. For phage titering, the strain TuD43 was used. Due to the presence of other prophages in given *P. aeruginosa* strains and their potential production, the sensitivity of filamentous phages to chloroform was used to accurately determine the total number of precipitated Pf phages. In parallel, the SPOT method was applied without and with chloroform treatment of the phage suspensions for 3 h (v/v 1:3). The incubation with chloroform was deliberately prolonged to certainly destroy Pf phage particles although generally filamentous phages can be permanently damaged by chloroform in a much shorter time (Griffith et al., 1981). The treated and untreated suspensions were tenfold diluted and tested in triplicate and in at least three independent experiments. Phage titer was determined using both double- and single-layer agar techniques. For the double-layer method, 3 ml of λ top (0.65% of agar) inoculated with 100 μL of TuD43 was added onto LB agar, containing 1.5% of agar. For a single layer, only 10 mL of identically inoculated λ top was used. Onto the λ top agar, 10 μl of phage dilutions was added, plates were incubated at 37°C and checked for plaque appearance after 8 and 24 h.

The total number of Pf phages (N_f_) was calculated as differences between the total numbers of phages obtained without chloroform treatment (N_T_ = filamentous + tailed phages) and the total number of phages after chloroform treatment (tailed phages; N_t_): N_f_ = N_T_-N_t_.

### CsCl Purification of Propagated Phages

After propagation by method 1 or 2 and precipitation with PEG8000, Pf bacteriophages were purified by equilibrium density gradient centrifugation [0.375 g/mL Cesium chloride, Sigma-Aldrich (CsCl)] at 135,000 × *g*, for 42 h at 4°C (Ti50 rotor, Beckman, United States). After ultracentrifugation, the formed bands in tubes were aspirated using syringe and needle, and phage suspensions were dialyzed in SM buffer (2 L of SM buffer for each milliliter of phage suspension).

### Phage Quantification in CsCl Purified Suspensions

To compare phage yield using these two methods, phage titer was determined in the dialyzed suspensions from both methods by the SPOT method. Tenfold dilutions of phage suspensions were prepared in SM buffer, ranging from 10^–1^ to 10^–8^. From each dilution, 10 μL were added on TuD43 lawn and incubated at 37°C for 24 h. After incubation, the plates with the single-layer SPOT method applied, plaques were counted at the spots at which the phage suspensions were added.

### ssDNA Isolation and Quantification

To isolate viral DNA, the dialyzed phages from both methods were treated with DNase and RNase overnight (5 U mL^–1^ and 10 μg mL^–1^). The nucleases were then inactivated at 65°C for 1 h, and EDTA, proteinase K, and SDS were added to phage samples to final concentrations of 20 mM, 50 μL/mL, and 0.5%, respectively. Samples were incubated at 56°C for 1 h and then cooled to room temperature. The standard phenol-chloroform procedure was used to isolate DNA, which was precipitated with double volume of 96% ice-cold ethanol and washed with 70% ice-cold ethanol. The supernatant was removed, and the dried pellet was dissolved in 30 μl of Elution buffer (MiniPrep, 10 mM Tris–HCL, pH 8.5). After phenol-chloroform isolation, DNA was subjected to 0.7% agarose gel electrophoresis and documented. The concentration of ssDNA was determined by BioSpec-nano (Shimadzu Biotech, Japan).

## Results

**FIGURE 2:**
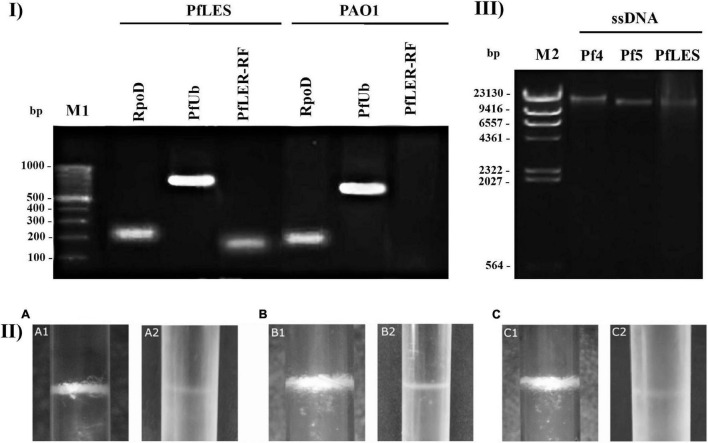
Confirmation of Pf phage production and method 2 advantage: **(I)** Agarose gel electrophoresis (0.7%) of PCR products that confirm PfLES RF production; M1 – 100 bp DNA ladder; **(II)** Pf bacteriophage bands after equilibrium density ultracentrifugation in CsCl (135,000 × *g*, 4°C, 42 h). The phage bands obtained using method 2 (1) are more distinctive than using method 1 (2); it was observed for all three phages: Pf4 **(A)**, Pf5 **(B)**, and PfLES **(C)**; **(III)** Agarose gel electrophoresis (0.7%) of ssDNA isolated from Pf virions with expected genome size of 12437 kb for Pf4, 10675 kb for Pf5, and 10569 kb for PfLES; M2 – Lambda HindIII ladder.

All three phages were successfully propagated using two methods and purified by CsCl equilibrium density gradient ultracentrifugation. It is known from the literature that Pf4 and Pf5 produce virions, whereas similar confirmation is absent for PfLES. Here, we confirmed the existence of PfLES RF (Figure 2-I), which implicates production of virions by this prophage. The expected product of RF was obtained for total LESB58 DNA although lacking in PAO1 as expected. Both the housekeeping gene and universal genetic element of the Pf phages are detected in DNA of both strains. The obtained RF sequence for PfLES (MG783392.1) confirmed previously predicted prophage ends, whereas other steps of the experiment confirmed both the virion and plaque production.

**FIGURE 3:**
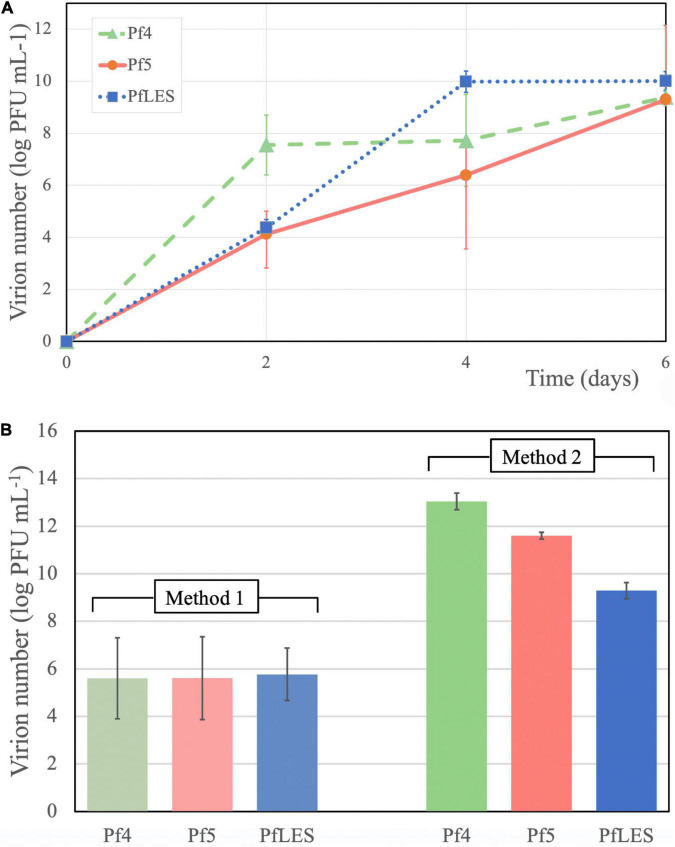
Pf phage yield in supernatants after 6 days of incubation and PEG8000 precipitation **(A)** and phage number after CsCl purification after application of two methods **(B)**.

The phage number increase of Pf4, Pf5, and PfLES during 6 days was monitored after PEG8000 precipitation. Chloroform treatment showed that such suspension can still contain tailed phages, as, for instance, after 2 days incubation, filamentous phages represent 50.8% of total phage number in PAO1 supernatant and 85.5% in UCBPP-PA14 (Table 2). According to Figure 3A, phage titers after days 2 and 4 significantly vary depending on Pf phage strain/bacterial strain, from 10^4^ to almost 10^8^ PFU mL^–1^. After 6 days, for all Pf phages, titers were in a range 10^9^–10^10^ PFU mL^–1^, which is acceptable for further phage purification.

**TABLE 2:** Proportion of filamentous phages in total phage number of PEG8000 precipitates.

PEG8000 precipitated *P. aeruginosa* supernatant	Days	Filamentous phage plaques in total plaques number (%)
PAO1	2	50.8
	4	60.3
	6	49.8
UBCPP-PA14	2	85.8
	4	88.5
	6	96.3

The bands of CsCl purified particles obtained after method 2 propagation were significantly thicker for all three phages (Figure 2-II). In the aspirated and dialyzed phage bands, number of virions was significantly higher after method 2 application as shown in Figure 3B. Phage titer varied between 10^5^ and 10^6^ PFU mL^–1^ after method 1, but after method 2, it varied between 10^9^ and 10^13^ PFU mL^–1^. Similarly, ssDNA yield after nucleic acid isolation from virions after method 1 was in the range 2.94–3.33 ng mL^–1^, whereas using method 2, much more ssDNA was obtained, ranging from 25.3 to 65.93 ng mL^–1^, depending on phage (Table 3). The isolated DNAs from virions corresponded to the expected genome size: approximately 12.5 kb for Pf4, and 10.5 kb for Pf5 and PfLES (Figure 2-III).

**TABLE 3:** Concentration of isolated ssDNA from CsCl purified Pf phages propagated using two methods.

	ssDNA (ng mL^–1^)		
	Pf4	Pf5	PfLES
Method 1	3.13 ± 0.2	2.94 ± 0.2	3.33 ± 0.8
Method 2	43.8 ± 4.9	65.93 ± 1.6	25.3 ± 1.8

**FIGURE 4:**
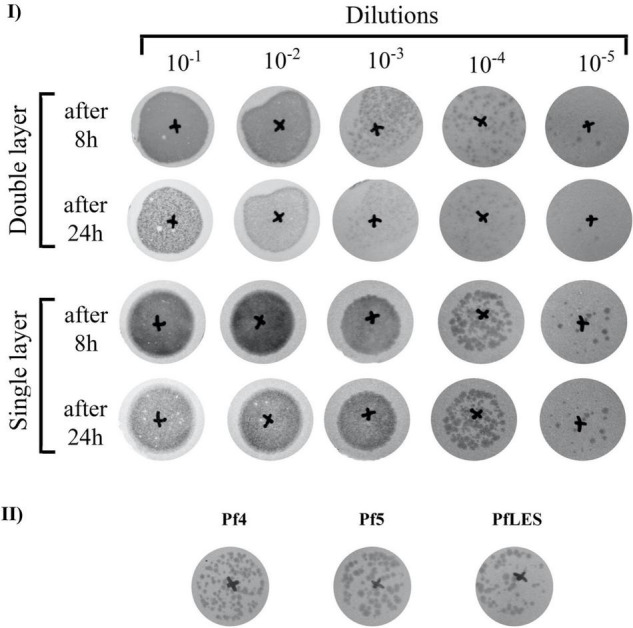
Filamentous phage plaque morphology on TuD43 *P. aeruginosa* lawns: **(I)** Tenfold dilutions of PEG8000 concentrated Pf4 virions after 6 days of incubation, using double- and single-layer SPOT method; **(II)** Comparative plaque morphology of three Pf phages.

We also noticed a phenomenon when the single- or double-layer SPOT method was applied for Pf phage titering. Namely, plaques were visible using both techniques after 8 h of incubation, but after 24 h, plaques on the double layer faded and became almost invisible (Figure 4-I). In this study, we confirm for the first time that both Pf5 and PfLES phages produce plaques on bacterial lawns (Figure 4-II). The plaques are small (∼1 mm) and turbid.

## Discussion

Filamentous phages of *P. aeruginosa* are not as productive as tailed phages during the lytic cycle. For instance, it was determined by qPCR method that only three Pf4 particles were produced per 10,000 cells in the lungs of mice after 48 h of infection (Secor et al., 2017). Due to the generally low production of filamentous phages, particularly the integrative representatives, we developed two methods to propagate and concentrate them. Method 1 favors a high volume of bacterial culture (6 L) with shorter incubation (48 h), and method 2 utilizes a smaller culture volume (600 mL) with prolonged incubation (6 days). All monitored parameters showed that method 2 is superior to method 1, and after application of method 2, we noticed that (a) phage bands after CsCl purification were more visible, which allowed easier aspiration; (b) the number of virions in the purified phage suspension after propagation by method 2 was from 3.5 to 7.4 logs greater; and (c) concentration of ssDNA isolated from virions were from 7.6 to 22.4 times greater. The production of phage particles over time increased from days 2 to 6 for approximately 5.6 logs for PfLES phage, 5.2 logs for Pf5 phage, and for 1.8 log Pf4 phage. It should be emphasized here that filamentous phages can be produced along with tailed phages present in a form of prophages in bacterial strains, and if they are not purified in CsCl suspensions, probably contain both filamentous and tailed phages (Smith and Scott, 1993; Boulnager, 2009; Mauritzen et al., 2020; Knezevic et al., 2021). Thus, it is crucial to determine total phage number, and then to treat suspensions with chloroform because filamentous phages are sensitive to this organic solvent and subsequently determine number of tailed phages. The precise number of filamentous phages can be calculated only by subtracting these values.

In both methods, the strains were incubated with agitation for two reasons: (1) To prevent stationary biofilm growth, including formation of surface pellicle, as phages can be trapped in the biofilm matrix, contributing to its structure (Secor et al., 2016a) and (2) To increase the chances for bacteriophages to adhere to host cells and to potentially superinfect the strain as superinfection leads to enhanced phage production (Rice et al., 2009). As the number of repetition steps increased, the viscosity of the medium with phages also increased. Although it seems to be a shortfall of the method, indeed it was previously confirmed that production of Pf phages is stimulated in viscous environments (Yeung et al., 2009). Moreover, upon the centrifugation step, although the supernatant was amended with fresh medium to obtain a starting volume of 600 mL (approximately added 100–200 mL) and regrowth was obvious, the nutritive conditions are still limited. Such nutrient-depleted conditions can additionally stimulate Pf phage production as previously confirmed for phage Pf5 (Lee et al., 2018). Finally, in method 2, several cycles of bacterial culture centrifugation were applied to avoid filtration because it was proven that filtration can significantly decrease filamentous particle number with recovery less than 0.01% (Piekarowicz et al., 2020). This is probably the reason why even Pf4 phage titer in CsCl purified suspensions (approximately 10^5^ PFU mL^–1^) was lower than in the original centrifuged and PEG precipitated stocks after 48 h (approximately 10^7^ PFU mL^–1^). The omitted filtration step in method 2, replaced with multiple culture centrifugation to remove bacterial cells, provided expected results, i.e., suspensions without bacterial contamination, appropriate for further steps of virion concentration and purification.

In method 1, the culture volume is 10 times greater (6 L) in comparison with method 2 (600 mL). The 10 times decreased volume is also an advantage of method 2, but incubation lasts 4 days longer, which can be considered a shortfall. However, significantly higher phage titer justifies the application of method 2. It is obvious that Pf4 reaches high titer in 2 days, PfLES in 4 and Pf5 in 6 days. Thus, 6 days of incubation is recommended for isolation of an unknown filamentous phage if its host is fast-growing, but prolongation of incubation should be considered when the host is a slow-growing bacterium.

The difference in plaque appearance using the single- and double-layer technique was observed in all three phages. Used λ top medium in both methods contained 0.65% of agar, so the difference could not be simply ascribed to the variation in agar content. It was indicated that, in filamentous phages, plaques are rather a result of decreased bacterial growth than true cell lysis, so probably the bottom part (LB agar) in the double layer method additionally supports bacterial growth. As a result of better bacterial growth, the plaques are paler and fade over time. Based on the observation, to precisely determine the filamentous phage number, we recommend Pf plaque counting after 8 h when double layer is applied and 8–24 h when a single layer is applied.

Finally, here, we confirm, for the first time, that PfLES can be produced from LESB58 strain and can form plaques. This finding implicates possible involvement of filamentous phages in virulence of the Liverpool epidemic cystic fibrosis *P. aeruginosa* strain, which should be determined in future studies. Just to notice, plaque formation by Pf5 was detected in this study for the first time.

Further development of method 2 is possible. For instance, it is possible to vary incubation temperature, medium content, or to expose infected bacterial hosts to various agents that can enhance phage production (e.g., antibiotics and UV irradiation). Similarly, the contribution of each part of the method can be further examined in detail (agitation, 0.45 and/or 0.22 μm filters application, variation of PEG8000 and/or NaCl concentration, etc.). It was previously shown that the phage Pf4 production is enhanced by anaerobic atmosphere (Platt et al., 2008), so additional improvement of the method for filamentous *Pseudomonas* phages propagation can be to seal flasks to favor anaerobic conditions. The method can be adapted for phages for which *P. aeruginosa* is not the host bacterium, but any other bacterium with modifications that are in line with host requirements. The newly optimized method 2 for propagation of Pf filamentous bacteriophages is simple, reliable, and economical due to lower volumes of used bacterial suspension. It is also appropriate for isolation of other phages that establish chronic productive infections even for filamentous phages that do not produce plaques. Finally, the described protocol is adjusted for medium-scale phage production but easily can be adopted for small or large scale if necessary.

## Data Availability Statement

The datasets presented in this study can be found in online repositories. The names of the repository/repositories and accession number(s) can be found below: https://www.ncbi.nlm.nih.gov/genbank/, MG783392.1.

## Author Contributions

Both authors listed have made a substantial, direct, and intellectual contribution to the work, and approved it for publication.

## Conflict of Interest

The authors declare that the research was conducted in the absence of any commercial or financial relationships that could be construed as a potential conflict of interest.

## Publisher’s Note

All claims expressed in this article are solely those of the authors and do not necessarily represent those of their affiliated organizations, or those of the publisher, the editors and the reviewers. Any product that may be evaluated in this article, or claim that may be made by its manufacturer, is not guaranteed or endorsed by the publisher.
